# AHD: Arabic healthcare dataset

**DOI:** 10.1016/j.dib.2024.110855

**Published:** 2024-08-22

**Authors:** Nashwan Ahmed Al-Majmar, Hezam Gawbah, Akram Alsubari

**Affiliations:** a Department of CS and IT, Faculty of Science, Ibb University, Yemen; b Department of Computers, Aljazeera University, Yemen

**Keywords:** Deep learning, NLP, Arabic healthcare dataset, AHD, Arabic healthcare, Medical assistant, Chatbot, Healthcare text classification

## Abstract

With the soaring demand for healthcare systems, chatbots are gaining tremendous popularity and research attention. Numerous language-centric research on healthcare is conducted day by day. Despite significant advances in Arabic Natural Language Processing (NLP), challenges remain in natural language classification and generation due to the lack of suitable datasets. The primary shortcoming of these models is the lack of suitable Arabic datasets for training. To address this, authors introduce a large Arabic Healthcare Dataset (AHD) of textual data. The dataset consists of over 808k questions and answers across 90 categories, offered to the research community for Arabic computational linguistics. Authors anticipate that this rich dataset would make a great aid for a variety of NLP tasks on Arabic textual data, especially for text classification and generation purposes. Authors present the data in raw form. AHD is composed of main dataset scraped from medical website, which is Altibbi website. AHD is made public and freely available at http://data.mendeley.com/datasets/mgj29ndgrk/5.

Specifications Table

**Table:** 

Subject	Computer Science, Data Science, Health and medical sciences.
Specific subject area	Arabic Language, Machine Learning, Health Informatics, Natural Language Processing, Text Generation, Text classification.
Type of data	Text/String
Data Format	Raw
Data collection	The dataset was collected from the Altibbi website using web-scraping tools. Python, with the Requests and BeautifulSoup packages, was used to collect the data.
Data source location	Primary Data Source: http://Altibbi.com/
Data accessibility	Repository name: Mendeley Data. Data identification number: 10.17632/mgj29ndgrk.5 Direct URL to data: http://data.mendeley.com/datasets/mgj29ndgrk/5

## 1 Value of the Data

• AHD is the largest, to our knowledge, available and representative Arabic Healthcare Dataset (AHD) for a wide variety category.
• AHD offers up to ninety distinct categories, making it robust for accurate text categorization.
• AHD offers over 808k distinct questions and answers, making it robust for accurate healthcare systems and chatbots.
• In contrast with the few small available datasets, AHD's size makes it a suitable corpus for implementing both classical as well as deep learning models.

## 2 Background

The progress of Natural Language Processing (NLP) is not significant in Arabic Language. To bring this progress introducing large datasets and research methodology should be emphasized. Therefore, authors have constructed a large Arabic Healthcare Dataset (AHD). The main objective of AHD is to contribute to healthcare system and Chatbots. AHD is created from Arabic content, which can help to develop practical in Arabic healthcare.

## 3 Data Description

Numerous language-centric research on healthcare is conducted day by day. To address shortcomings of Arabic natural language generation models, authors introduce a large Arabic Healthcare Dataset (AHD) of textual data. For this motivation, authors named our dataset ‘AHD’ [3].

The largest Arabic Healthcare Dataset (AHD) as we know was collected from medical website. The AHD consists of more than 808k rows 90 variety categories AHD adopted the annotation of each question as it appeared on its source website, Altibbi. Table 1 summarize distribution of question and answer per category.

**Table 1:** Distribution of question and answer per category.

No	Category	Size	Ratio	No	Category	Size	Ratio
1	Gynaecological	162,142	20.06	46	Herbalists	402	0.05
2	Sexual health	96,398	11.92	47	Urology	392	0.05
3	Musculoskeletal and joint	45,826	5.67	48	Alternative medicine	387	0.05
4	Urinary and reproductive tract	42,599	5.27	49	Infertility	386	0.05
5	skin	41,760	5.17	50	psychology	382	0.05
6	General Medicine	41,740	5.16	51	Gynaecological surgery	260	0.03
7	Gastrointestinal	36,554	4.52	52	Diagnosis	256	0.03
8	Paediatric	23,818	2.95	53	Hormones	240	0.03
9	Sexual	22,165	2.74	54	laboratory	224	0.03
10	Cardiovascular disease	22,013	2.72	55	Genetic Disease	166	0.02
11	Psychiatric illness	19,091	2.36	56	Vitamins and minerals	150	0.02
12	Pregnancy and Birth	18,763	2.32	57	Immunology	92	0.01
13	eyes illnesses	18,682	2.31	58	Radiology	83	0.01
14	Nose, ear and throat	18,674	2.31	59	optics	82	0.01
15	Esoteric	18,123	2.24	60	medical services	77	0.01
16	General Surgery	17,348	2.15	61	First aid	68	0.01
17	Dental	16,901	2.09	62	Embryology	66	0.01
18	feed	16,344	2.02	63	Anatomy	61	0.01
19	Pharmacology	14,890	1.84	64	Pathology	57	0.01
20	Malignant and benign tumours	12,361	1.53	65	to drug	56	0.01
21	Neurological	10,845	1.34	66	toxicology	55	0.01
22	Psychological health	10,349	1.28	67	biology	49	0.01
23	Endocrine	9237	1.14	68	Microbiology	48	0.01
24	Child health	8270	1.02	69	Genetics	44	0.01
25	diabetes	7147	0.88	70	Physiology	34	0.00
26	Skin and beauty	6656	0.82	71	Preventive Medicine	34	0.00
27	respiratory system	6102	0.75	72	Rheumatic	34	0.00
28	Hypertension	4557	0.56	73	Vaccines and vaccinations	32	0.00
29	Oral	4411	0.55	74	Birth Defect	21	0.00
30	Orthopaedic Surgery	4194	0.52	75	chemistry	18	0.00
31	Teeth health	3967	0.49	76	History of medicine	18	0.00
32	Men's health	3806	0.47	77	Elderly health	17	0.00
33	Women's health	3608	0.45	78	Paediatric surgery	16	0.00
34	Blood	2362	0.29	79	Ramadan	13	0.00
35	public health	2307	0.29	80	Biochemistry	12	0.00
36	dentist	2232	0.28	81	Vascular surgery	12	0.00
37	addiction	1915	0.24	82	Carry tubes	12	0.00
38	Plastic surgery	1579	0.20	83	Medical equipment	8	0.00
39	Health and sports	1114	0.14	84	physics	8	0.00
40	Jaw and dental surgery	685	0.08	85	Metabolic	7	0.00
41	Neurosurgery	593	0.07	86	Botany	5	0.00
42	Cardiovascular surgery	575	0.07	87	Histology	5	0.00
43	Allergic allergy	555	0.07	88	Medical News	2	0.00
44	physical therapy	418	0.05	89	organic chemistry	1	0.00
45	Infectious	403	0.05	90	Ecology	1	0.00

**Fig. 1:**
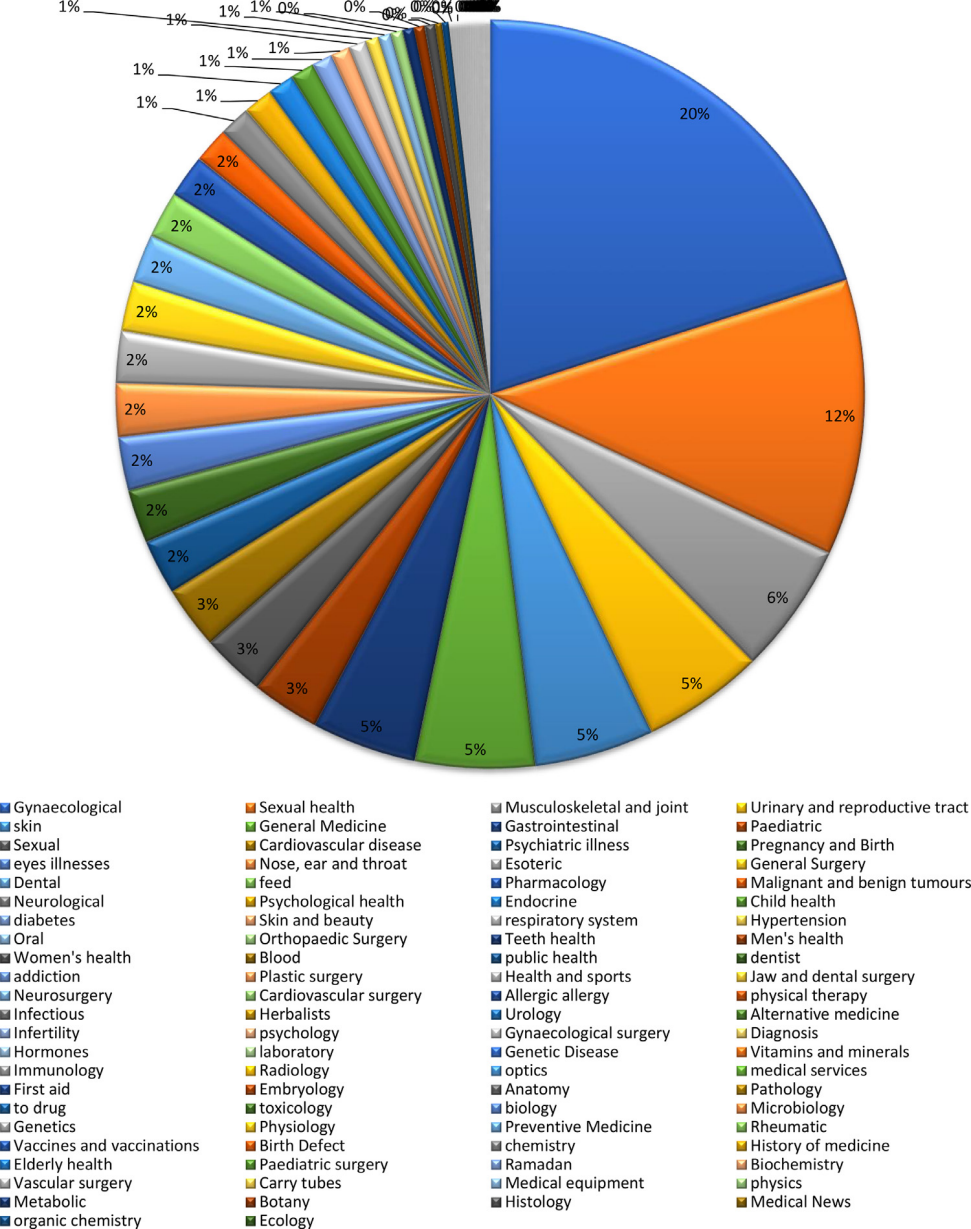
Distribution of question and answer per category.

Fig. 1 show the distribution of question and answer per category.

The questions and answers in Arabic Healthcare Dataset (AHD) have different lengths. The average length (Average number of characters) of the questions and answers are 115, and 152, respectively. Authors also figured out the maximum and minimum word count of the Arabic Healthcare Dataset (AHD). The maximum characters for questions and answers are 348, and 32,767, and the minimum is respectively 3, and 2. Besides character counts, authors identify the word counts also. All of these pieces of information are mentioned in Table 2. These pieces of information are determined from raw data.

**Table 2:** All numeric information for Arabic Healthcare Dataset (AHD).

	Total	Maximum Word count	Minimum Word count	Maximum Character count	Minimum Character count	Average Numbers of Words	Average Numbers of Character
Questions	808,472	32	1	348	3	21	115
Answers	808,472	5905	1	32,767	2	26	152

The data is kept in raw format as excel; no cleaning, stemming or any type of pre-processing is applied after scraping. The AHD contain some English symbols, punctuation, digits, and almost no Arabic diacritics.

Table 4 shows sample of Arabic Healthcare Dataset (AHD).

AHD.xlsx provides the raw data include healthcare questions, answers and categories in Arabic.

AHD_englishe.xlsx provides raw data that includes questions, answers, and health care categories translated from Arabic to English.

## 4 Experimental design, Materials and Methods

Arabic texts may exhibit a scarcity of healthcare. To address this problem and to facilitate the training of natural language generation models on correct Arabic healthcare texts, it is necessary to construct a large dataset that is dedicated to Arabic healthcare.

**Fig. 2:**
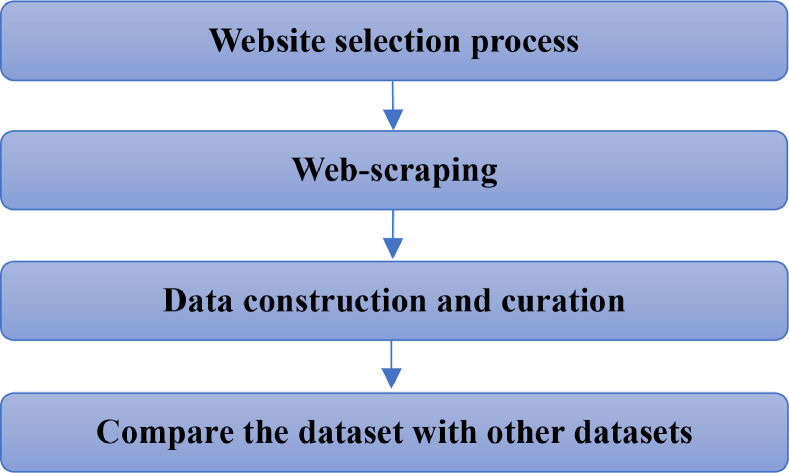
Experimental design, materials and methods.

Fig. 2 shows Experimental design, materials and methods.

• Website selection process

The website selection process required the following conditions to be met:

- The website must be specialized in medicine, especially healthcare.
- The website must be used mainly in Arabic and not as a translation into Arabic.
- The website should allow membership only for specialists who are experts in the medical field, such as doctors, nurses, and pharmacists, after reliably proving their experience.
- The website should allow questions to be answered only by members.

The authors choose the Altibbi website [2] after examining it and ensuring that it fulfills the previous conditions.

• Web-scraping

The dataset was retrieved from websites using web-scraping tools, as well as Python, which has many packages, including Requests and BeautifulSoup, which support the retrieval of data from the web, authors utilized Google Colab, Google's cloud-based notebook.

Requests is an integral Python module for handling HTTP requests, using methods such as GET, POST, DELETE, and HEAD. The GET method was used to retrieve HTML pages from the specified sources. In addition, BeautifulSoup, another package used, can extract information from HTML pages, but the only information required from this dataset is the question, answer and category.

• Data construction and curation

In the collection process, several criteria were considered to retrieve data from medical website (Altibbi), as follows:

• The medical website is retrievable, as some websites strictly unretrievable.
• The structure of the medical website is based on pages that loop according to date.
• The website medical addresses one of the targeted categories.
• Compare the dataset with other datasets

Authors kept dataset in raw format. No cleaning, stemming or any type of pre-processing is applied after scraping. AHD contains some English symbols, punctuation, digits, and almost no Arabic diacritics.

In Table 6, a comparison between the AHD from Arabic Healthcare Question and Answer, along with other datasets described in the relevant literature (Abdelhay et al., 2023) [1].

Also, Table 6 shows a comparison between our dataset (AHD) and the other datasets, which indicates that AHD dataset is the largest Arabic dataset in the healthcare domain. AHD can be used for several tasks, such as text classification or text generation.

Tables 3 and 5 shows sample of Arabic Healthcare Dataset (AHD) which translated to English [3].

**Table 3:** All numeric information for Arabic Healthcare Dataset (AHD) in translated to English.

	Total	Maximum Word count	Minimum Word count	Maximum Character count	Minimum Character count	Average Numbers of Words	Average Numbers of Character
Questions	808,472	112	1	658	1	31	166
Answers	808,472	5905	1	32,767	1	34	203

**Table 4:** Sample reading comprehension of the Arabic Healthcare Dataset (AHD).

**Table 5:** Sample reading comprehension of Arabic Healthcare Dataset (AHD) in translated to English.

Questions	Answers	Category
A heart rate of 98 is normal for a 54-year-old person with diabetes	We recommend careful planning and effort	Cardiovascular disease
Pus cells: My urine test result is 40 to 50. What treatment is required?	Do a urine culture to determine the type of antibiotic Drink plenty of water and fluids Epimag effervescent twice for five days	Urinary and reproductive tract diseases
I am pregnant for the first time in the tenth week and I have pain similar to menstrual pain. Is this normal?	Yes, normal	Gynecological diseases

**Table 6:** Comparison Arabic Healthcare Dataset (AHD) with other datasets.

Dataset	Task	Size
MAQA (Abdelhay and Mohammed 2022)	MedicalBot.	430,000
ASMCHA (Alayba et al. 2017)	Sentiment analysis.	126,959
Arabic empathetic dialogues (Naous et al. 2021)	Empathetic bot.	36,628
Private dataset (Habib et al. 2021)	Medical recommendations.	36,628
DZchatbot (Boulesnane et al. 2022)	Chatbot.	81,659
Corpus on Arabic Egyptian tweets (Kora and Mohammed 2019)	Sentiment analysis.	50,000
**AHD: Arabic Healthcare Dataset**	**Text classification, Chatbot, Question answering,** **Word embedding.**	**808,472**

## Limitations

There are several limitations to the AHD that need to be acknowledged. Firstly, the AHD was collected from a one website. Secondly, the AHD is unbalanced, as some categories contain a large number of questions and answers, while some categories contain a small number of questions and answers.

## Ethics Statement

Terms of Service (ToS): Authors have considered and followed the source website Altibbi's ToS, privacy laws, and user consents.

Copyright: The authors have read and followed the ethical requirements for publication in Data in Brief and confirmed that the current work does not involve any type of human studies, animal studies, or data gathered using social media. All data belongs to the source website Altibbi through user consent, and it's open to the public, so ethical approval has not been sought. The data adopted the annotation of each question and answer as appeared on the source website Altibbi and the distribution of data per category. We can confirm that this manuscript adheres to ethical publishing standards.

Privacy: The authors have anonymized all participant data and confirm that all the data is non-sensitive.

Scraping policies: There are not specific scraping policies for the source website Altibbi.

## CRediT Author Statement

**Hezam Gawbah:** Conceptualization, Methodology, Data construction, Data curation, Visualization, Soft-ware, Investigation, Writing – original draft; **Nashwan Ahmed Al-Majmar:** Conceptualization, Supervision, Writing – review & editing; **Akram Alsubari:** Conceptualization, Supervision, Writing – review & editing.

## Data Availability

AHD: Arabic Healthcare Dataset (Original data) (Mendeley Data).
